# Rural and urban differences in blood pressure and pregnancy-induced hypertension among pregnant women in Ghana

**DOI:** 10.1186/1744-8603-9-59

**Published:** 2013-11-14

**Authors:** Deborah van Middendorp, Augustinus ten Asbroek, Fred Yaw Bio, Anthony Edusei, Lyonne Meijjer, Sam Newton, Charles Agyemang

**Affiliations:** 1 Department of Public Health, Academic Medical Centre, University of Amsterdam, Netherlands PO Box 22660, Amsterdam, The Netherlands; 2 Department of Population Health, Faculty of Epidemiology and Population Health, London School of Hygiene and Tropical Medicine, London, UK; 3 Department of Community Health, School of Medical Sciences, Kwame Nkrumah University of Science and Technology, Kumasi, Ghana; 4 Kintampo Health Research Centre, Kintampo, Ghana

**Keywords:** Blood pressure, Pregnancy induced hypertension, Pregnancy, Ghana

## Abstract

**Background:**

Globally, about 350.000 women die every year from pregnancy related causes and more than half of these deaths occur in sub-Saharan Africa (SSA). Approximately 12% of the maternal deaths are associated with hypertensive disorders in pregnancy such as pregnancy induced hypertension (PIH). However, very little is known about PIH and associated determinants in many SSA countries such as Ghana. We therefore sought to assess rural and urban differences in blood pressure (BP) and PIH among pregnant women in Ghana.

**Methods:**

We conducted a cross-sectional study among 967 rural (677) and urban (290) pregnant women with a gestational age of more than 20 weeks. PIH was defined as a systolic blood pressure of ≥140 mmHg and/or diastolic blood pressure of ≥90 mmHg.

**Results:**

Women in urban Ghana had a higher mean systolic and diastolic BP than women in rural Ghana (105/66 mmHg versus 102/61 mmHg, p < 0.001 for both systolic and diastolic BP). The prevalence of PIH was also higher in urban Ghana (3.1%) than in rural Ghana (0.4%) (p = 0.014). The urban and rural difference in mean diastolic blood pressure persisted even after adjustments for the study characteristics in a linear regression model. In both rural and urban Ghana, BMI, heart rate and a family history of hypertension were independently associated with BP.

**Conclusion:**

Our findings suggest higher mean BP levels and PIH in urban Ghana than in rural Ghana. BMI was independently related to high BP. Left unchecked, the increasing prevalence of overweight and obesity in Ghana will exacerbate PIH levels in Ghana.

## Background

The fifth goal of the United Nations' Millennium Development Goals for 2015 is to reduce the maternal mortality ratio (MMR) by three fourths [1]. This is because about 350.000 women die every year from pregnancy related causes globally [2]. Evidence also shows huge differences in MMR between rich and poor world regions. In poor resource regions of the world, the MMR was 450 maternal deaths per 100 000 live births, compared with 9 maternal deaths per 100 000 live births in rich regions in 2008. Sub-Saharan Africa (SSA) has been most affected with more than half of all these deaths [2]. Hypertensive disorders of pregnancy, haemorrhage, severe anaemia, sepsis, obstructed labour and unsafe abortion and its complications are the main direct causes of maternal death [3,4].

Pregnancy-induced hypertension (PIH) is a leading cause of maternal and perinatal mortality and can also lead to long-term health problems like chronic hypertension, kidney failure, or nervous system disorders [5,6]. PIH is a syndrome of hypertension with or without proteinuria, with the clinical manifestation usually occurring late in pregnancy and regressing after delivery. It is a major pregnancy complication, causing premature delivery, foetal growth retardation, abruptio placentae, and foetal death, as well as maternal morbidity and mortality. Approximately l0-15% of maternal deaths in low- and middle- income countries is associated with PIH [7,8].

Some SSA countries such as Ghana have made a significant progress in reducing MMR. For example, MMR was reduced by 23% between 2000 and 2008 in Ghana, but the rate (i.e. 350 per 100.000 live births in 2011) is still very high compared to high income countries [2]. Given the relatively high rate of MMR in Ghana, it is imperative to identify the risk factors of maternal deaths to help guide intervention initiatives [9]. Hypertension related disorders have been identified as an important cause of maternal death [10-12]. A 13-year hospital-based study in rural Ghana, for example, found that between 1987 and 2000 (pre-) eclampsia was responsible for 4.8% of the total maternal deaths [10]. In addition, in the Accra-metropolis during the year 2002, 14.6% of the maternal deaths were caused by eclampsia [12]. Although, hypertension disorders contribute importantly to maternal deaths, little is known about the factors that might underlie these disorders in Ghana. Studies in Ghana show important differences in BP and the prevalence of hypertension between urban and rural Ghana [13,14]. It is unclear, however, whether these observed rural and urban differences are also seen among pregnant women in Ghana.

Therefore the main objective of this study was to assess rural and urban differences in BP and PIH among pregnant women in Ghana; and to identify factors associated with high BP.

## Methods

### Study population and sampling

This comparative cross-sectional study was carried out among pregnant women who visited the selected antenatal clinics for a general check-up. Urban data were collected from the antenatal clinic of Kwame Nkrumah University of Science and Technology (KNUST) hospital in Kumasi (capital of the Ashanti region), and rural data were collected from antenatal clinics of government health facilities in Kintampo, Techiman, Nkoranza, Busunya, Akuma and Dawadawa in the Brong-Ahafo region of Ghana. These two regions share a boundary, and the environmental characteristics such as climate and diet are largely similar.

All pregnant women with a pregnancy of 20 weeks or more were identified through the midwifery/medical notes and included for the study. Gestational age in women attending KNUST was based on ultrasound findings, for all the other women gestational age was based on the midwifery notes only. The selected women were approached to participate in the study and were asked to sign a consent form before their measurements were taken. Pregnant women with a gestational age of less than 20 weeks were excluded from this study. Consecutive sampling was used to include the patients in the study upon registration at the antenatal clinic. The total number of women that were included was 967 of which 290 were included in urban Ghana and 677 in rural Ghana.

Data-collection in rural areas was coordinated from Kintampo Health Research Centre (KHRC). Data were collected from July to September 2008 in rural Ghana and between March and May 2010 in urban Ghana. Ethical approval was obtained from the KNUST Committee on Human Research, Publication and Ethics, and the Kintampo Health Research Centre Scientific Review Committee and the Kintampo Health Research Centre Institutional Ethics Committee.

### Measurements

Body weight was measured after removal of shoes, heavier clothes and pocket contents using a SECA 361 model weighing scale. Body height was measured to the nearest 1 cm after removal of the shoes. BMI was calculated as weight (kg) divided by height (m^2^). Overweight was defined as BMI ≥25 kg/m^2^ and obesity as BMI ≥30 kg/m^2^. Blood pressure was measured three times with a validated Oscillometric automated digital BP device (Omron M-6 comfort device), 1 minute apart in seated position with appropriate cuff sizes on the left arm by trained medical students and nurses. Omron M-6 comfort device has been validated and is recommended by various hypertension societies for measuring BP in pregnancy [15]. The mean systolic and diastolic BP of the two last readings were recorded and used for analysis. In addition, history of PIH was extracted from the midwifery/medical notes. Furthermore, information on age, marital status, pregnancy duration, parity, history of hypertension, antihypertensive medication, lifestyle (smoking and alcohol consumption) and socio-economic status were collected by a short questionnaire. PIH was defined according to the International Society for the Study of Hypertension (i.e. systolic BP ≥140 mmHg and/or diastolic BP ≥90 mmHg occurring after 20 weeks in a woman who was normotensive before 20 weeks gestation). Chronic hypertension was defined as BP ≥140/90 or receiving anti-hypertensive medication before the 20th week of pregnancy [16].

### Data analyses

Chi-square tests were used to assess differences in categorical variables. Continuous variables were analysed using an independent sample *t*-tests. Linear regression was used to identify independent factors associated with systolic and diastolic blood pressure. All statistical tests were two-tailed and *P*-values ≤ 0.05 were considered as statistically significant. All statistical analyses were performed using SPSS 17.0 for Mac OSX (SPSS Inc. Chicago, USA).

## Results

### Characteristics of the study population

Pregnant women in rural Ghana were less educated, less likely than their urban counterparts to have white-collar jobs, and were less obese, but had a higher parity than their urban peers. The prevalence of self-reported diabetes (0.7% versus 0.3% p = 0.327) and chronic hypertension (0.3% versus 0.4%, p = 0.870) were low in both urban and rural Ghana and the differences were non statistically significant. The prevalence rates of smoking (0.3% versus 0.1%, p = 0.538) and alcohol consumption were low and similar (1.7% versus 1.2%, p = 0.506) in both rural and urban groups. The difference in mean age was not statistically significant (Table 1).

**Table 1:** Characteristics of the study population

	***Urban (n=290)***	***Rural (n=677)***	
			p-value
**Age (mean and SD)**	27.8 (5.3)	27.3 (6.0)	0.281
**Parity n (%)**			<0.001
0	104 (35.9)	142 (21.0)	
1 - 4	181 (62.4)	425 (62.8)	
>=5	5 (1.7)	110 (16.2)	
**Educational level n (%)**			<0.001
None or primary school	45 (15.5)	214 (31.6)	
Junior secondary school/Middle school	118 (40.7)	221 (32.6)	
Senior secondary school or higher	125 (43.1)	129 (19.0)	
Unknown	2 (0.7)	113 (16.7)	
**Profession ion n (%)**			<0.001
Domestic worker/farmer/labourer	9 (3.1)	174 (25.7)	
Seamstress/hairdresser/trader/food seller	163 (56.2)	332 (49.0)	
Teacher/nurse/accounts/administrative/clerical/secretarial	64 (22.1)	10 (1.5)	
Other	24 (8.3)	2 (0.3)	
Unknown	2 (0.7)	159 (23.5)	
None	28 (9.7)	0 (0.0)	
**BMI (kg/m2)**	28.0 (4.8)	25.8 (4.0)	< 0.001
BMI categories n (%):			< 0.001
BMI <18.5	0 (0.0)	2 (0.3)	
BMI 18.5 - < 25	84 (29.0)	327 (48.3)	
BMI 25 - < 30	123 (42.2)	252 (37.2)	
BMI >30	83 (28.6)	91 (13.4)	
Unknown	0 (0.0)	5 (0.7)	
Systolic BP (mmHg)	104.8 (12.8)	101.9 (10.9)	<0.001
Diastolic BP (mmHg)	66.0 (9.6)	61.1 (7.7)	<0.001
Pulse rate	93.4 (11.8)	91.1 (11.9)	0.130
**Smoking n (%)**			0.538
Yes	1 (0.3)	1 (0.2)	
No	289 (99.7)	674 (99.6)	
Unknown	0 (0.0)	2 (0.2)	
**Alcohol consumption n (%)**			0.506
Yes	5 (1.7)	8 (1.2)	
No	285 (98.3)	667 (98.5)	
Unknown	0 (0.0)	2 (0.3)	

Values are mean (SD) or n (%). BMI, body mass index; BP, blood pressure.

### Pregnancy-induced hypertension and mean BP

**Figure 1:**
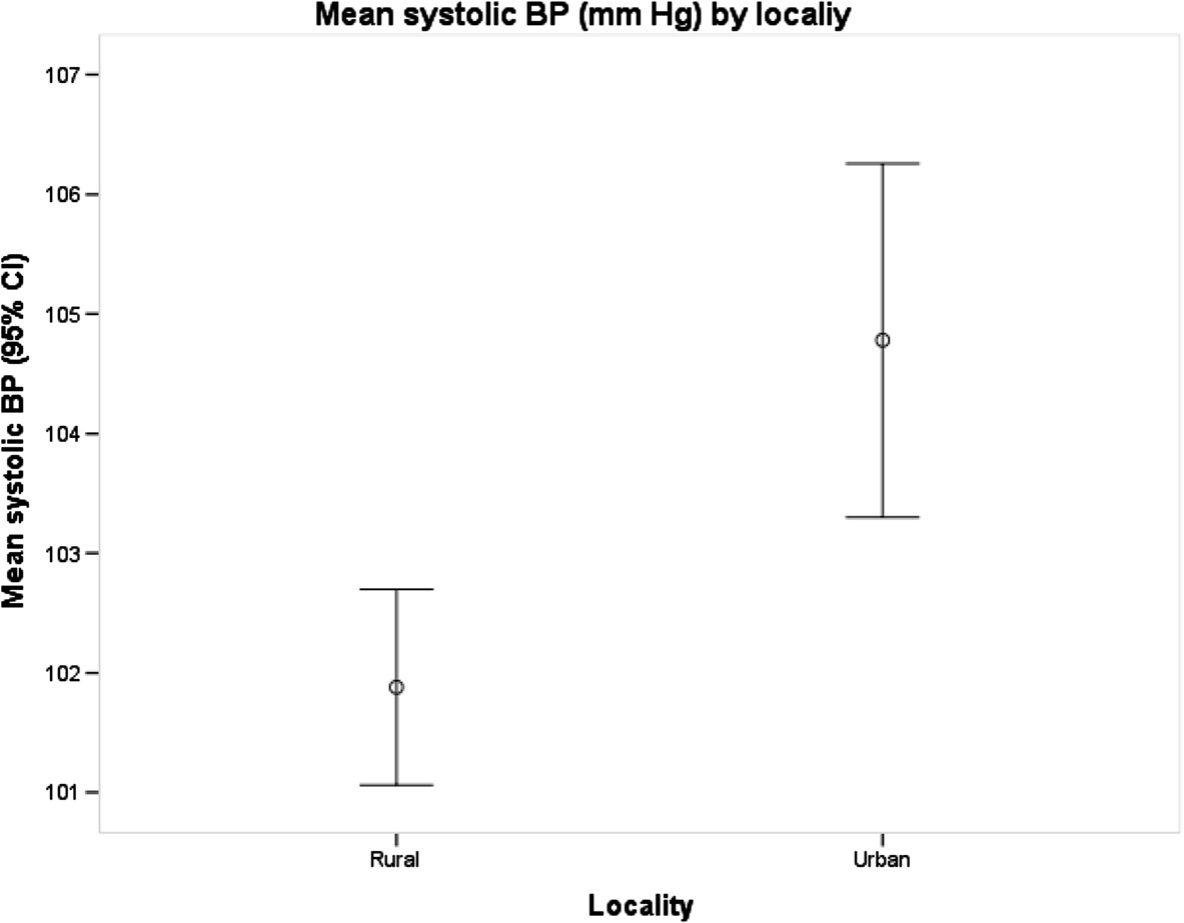
Mean systolic blood pressure by locality.

**Figure 2:**
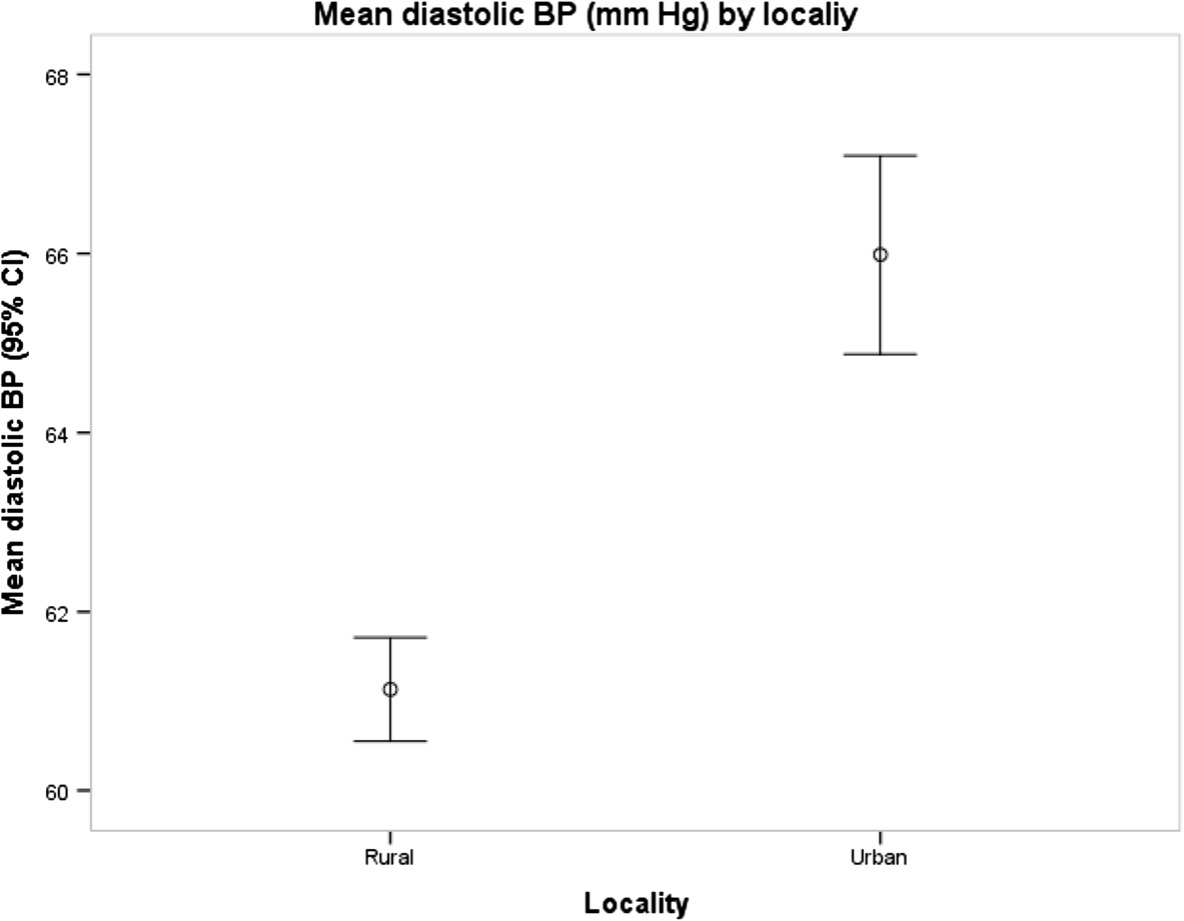
Mean diastolic blood pressure by locality.

**Figure 3:**
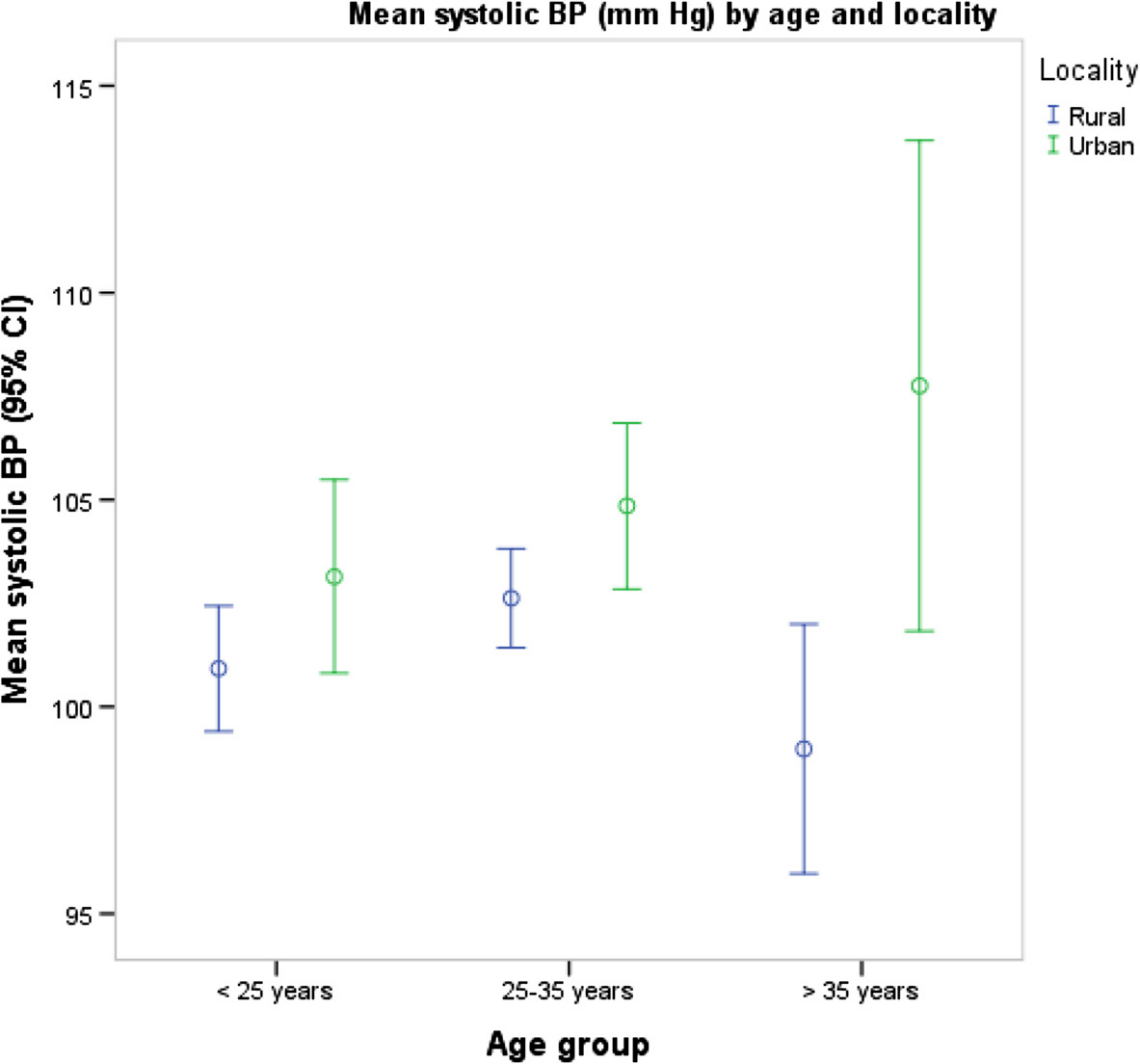
Mean systolic blood pressures by age group and locality.

**Figure 4:**
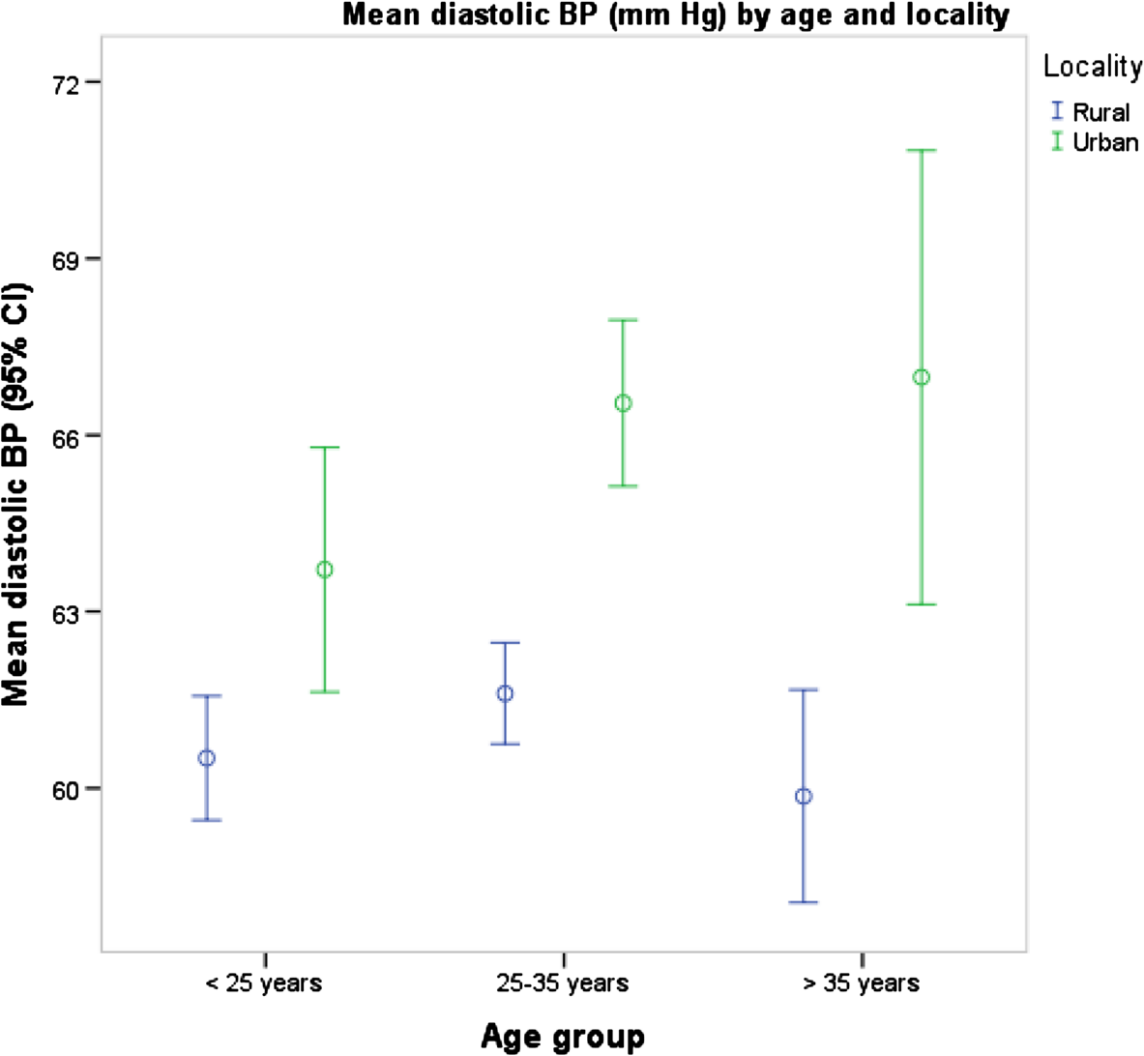
Mean diastolic blood pressures by age group and locality.

The prevalence of PIH was higher in urban women than in rural women (3.1% versus 0.4%, p = 0.014). The mean systolic BP (p < 0.001) and diastolic BP (p < 0.001) levels were also higher in urban Ghana than in rural Ghana (Figures 1 and 2) and in all age groups although the differences were more pronounced for diastolic BP than for systolic BP (p = 0.031) (Figures 3 and 4). Among urban women, diastolic BP (p = 0.015), and systolic BP to some extent (p = 0.084) increased with age. Among rural women, however, there were no relationship between BP and age (Figures 3 and 4). In a simple linear regression analysis, urban residence, BMI, family history, high education and white collar jobs were related to high systolic BP (Table 2). By contrast, parity was inversely related to low systolic BP. For diastolic BP, urban residence, age, BMI, heart rate, family history of hypertension, high education levels and white-collar jobs were positively related to high BP while parity was inversely related to BP. In multiple linear regression analyses, BMI and heart rate were the only factors that were independently related to high systolic BP; and only urban residence and BMI were independently related to diastolic BP (Table 2).

**Table 2:** Univariate and multivariate regression analysis of systolic and diastolic blood pressure by study characteristics

***Systolic blood pressure***	**Univariate**		**Multivariate**	
	**Beta**	**SE**	**Beta**	**SE**
Urban	2,899**	0.806	0.096	0.961
Age (linear)	0.075	0.070	-	-
BMI (linear)	0.551**	0.084	0.445**	0.088
Parity (linear)	-0.402*	0.181	-0.260	0.185
Heart rate (linear)	0.109**	0.032	0.091**	0.032
Family history of hypertension, yes	3.758**	1.155	2.524*	1.162
Junior secondary school/Middle school§	0.961	0.860	0.170	0.871
Senior secondary school or higher§	3.740**	0.933	1.508	1.033
Seamstress/hairdresser/trader§╪	2.101	0.771	1.036	0.788
Teacher/nurse/accounts/administrative/clerical/secretarial╪	6.257**	1.448	2.243	1.677
***Diastolic blood pressure***	**Univariate**		**Multivariate**	
	**Beta**	**SE**	**Beta**	**SE**
Urban	4.857**	0.583	2.933**	0.670
Age (linear)	0.122*	0.051	0.060	0.049
BMI (linear)	0.491**	0.062	0.341**	0.067
Parity (linear)	-0.281*	0.135	-0.018	0.152
Heart rate (linear)	0.185**	0.023	0.167**	0.023
Family history of hypertension, yes	4.443**	0.852	2.220**	0.841
Junior secondary school/Middle school§	1.423*	0.634	-0.156	0.660
Senior secondary school or higher§	4.164**	0.688	1.234	0.762
Seamstress/hairdresser/trader╪	1.156	0.554	0.063	0.599
Teacher/nurse/accounts/administrative/clerical/secretarial╪	6.836**	1.067	1.355	1.196

§Reference category is no education or primary school; ╪ Reference category is Domestic worker/farmer/labourer.

*=p<0.05 **=p<0.01.

## Discussion

Our study findings indicate that the mean BP and PIH levels were lower in rural Ghana than in urban Ghana. BMI and heart rate were independently related to a high systolic BP while urban residence and BMI were independently related to diastolic BP.

The low prevalence of PIH in rural (0.4%) and urban (3.1%) Ghana in our current study is more or less consistent with studies in SSA. In South Nigeria, a prevalence of 3.7% was found [17]. For example, in rural areas of Zimbabwe a prevalence of PIH of 1.8% was found [18]. Retrospective hospital based data also show low incidence rates in several African countries [19-21]. In one study in urban Gabon, a low incidence of 0.5% was found [19]. In urban Ethiopia an incidence of 0.7% was found over a study period of 5 years (1994 – 1999) [21]. Furthermore, a retrospective study in the Dar es Salaam found an incidence rate of 2% [22].

Although the prevalence rates in our study are somewhat consistent with studies in several African countries, the mean BP levels were extremely low in Ghana in both urban (105/66 mmHg) and rural (102/61 mmHg) areas when compared with other studies in SSA [23-26]. In Ethiopia, for example, women in the age group of 25–34 years had a mean BP of 117/77 mmHg while those in the age group of 35–44 years had mean BP of 122/79 mmHg [23]. Similarly, in urban Tanzania the age group of 15–34 had a mean blood pressure of 119.7/78.1 mmHg, and in rural Tanzania this age group had a mean blood pressure of 115.9/72.5 mmHg [24]. Furthermore, the mean blood pressure levels are far lower than those reported among non pregnant women with the same age range in Ghana. In one study in rural parts of Northern Ghana, the mean systolic and diastolic BP levels for the age group 15–24 years, 25–34 years and 35–44 years were 114/66 mmHg, 113/68 mmHg and 123/76 mmHg, respectively [25]. Usually the diastolic blood pressure decreases between 5 mmHg to 10 mmHg during week 12–26 of the pregnancy [26]. So the mean BP levels of 105/66 mmHg in urban and 102/61 mmHg in rural Ghana in this study are quite low compared to the previous found BP levels among women in these age groups. The explanations for the low mean BP levels among Ghanaian pregnant women are unclear and further studies are needed to establish the potential underlying factors.

In both rural and urban Ghana, BMI, heart rate and a family history of hypertension were independently associated with an increased BP. These findings are consistent with previous studies carried out in SSA [23,27-31]. For diastolic BP, the difference between rural and urban Ghana could be partially explained by a difference in mean BMI and number of women with a family history of hypertension, though another unknown factor might have contributed to the difference in diastolic BP.

BMI was the strongest factor for differences between urban and rural Ghana. In the multiple linear regression analysis, BP difference between urban and rural Ghana was partially explained by the difference in BMI. This observation is consistent with a recent report in China. In Liu *et al’s* study in China, the incidence of PIH tends to rise with an increasing BMI [26]. Overweight and obesity are an increasing problem in cities of Ghana. Left unchecked, the increasing prevalence of overweight and obesity in Ghana will exacerbate PIH levels in Ghana.

Some limitations were inherent to our study design. As in many surveys, the BP levels were based on the average of two measurements at a single visit, which might have overestimated the prevalence rates due to white-coat hypertension [32]. The urban sample was based on only one hospital in the Ashanti region, unlike the rural sample which was based on different health facilities in the Brong-Ahafo region. It is possible that the characteristics of the study population may differ from other urban hospitals and could influence our study results. Nevertheless, because it is located in the university grounds, it serves a population with a high degree of variation in socioeconomic status. Besides, the urban sample was relatively small.

The assessment of gestational age was based on last menstrual period recorded in midwifery notes in rural Ghana and ultrasound in urban Ghana, which might influence the our study results [33]. In addition, the urban and rural data collections were performed in different months of the year and therefore bias for timing might be present. TePoel et al. found a difference in prevalence of PIH between wet and dry seasons in tropical countries [34]. Nonetheless, data collection in both rural and urban Ghana was carried out mainly in rainy season (i.e. April to October), therefore the influence of seasonal differences in data collection is likely to be small.

Overweight and obesity measures in our study were based on BMI during pregnancy, which might not be a true reflection of overweight and obesity because of changes in weight during pregnancy [35]. In many studies pre-pregnancy BMI or weight gain during pregnancy was used to assess overweight or obesity. Unfortunately, since most pregnant woman in rural Ghana do not know their pre-pregnancy weight, it was not possible to assess this in our study. Caution is therefore needed regarding the interpretation of our result on this.

Despite these limitations, the current study still provides very important information on one of the important risk factors for poor maternal health outcomes.

## Conclusion

The study findings suggest higher mean BP levels and PIH in urban Ghana than in rural Ghana. BMI was independently related to high blood pressure. The increasing prevalence of overweight and obesity in Ghana will exacerbate PIH levels in Ghana if efforts are not taken to curb the situation.

## Abbreviations

MMR: Maternal mortality rates (MMR) are calculated as deaths per 100 000 births, extending from 28 weeks of pregnancy to 6 weeks postpartum; BMI: Body-mass-index. Defined as weight (kg)/ height (m)^2^; PIH: Pregnancy-induced hypertension; JSS: Junior secondary school; SSS: Senior secondary school; BP: Blood pressure; SBP: Systolic blood pressure; DBP: Diastolic blood pressure; ANC: Antenatal care.

## Competing interests

The authors declare that they have no competing interests.

## Authors’ contributions

DvM, CA, GtS & LM conceived the idea, developed and refined the methodological approach in collaboration with FY, AKE & SN. DvM carried out the statistical analysis and wrote the first draft. All contributed to the interpretation of the results. All authors read and approved the final manuscript.
